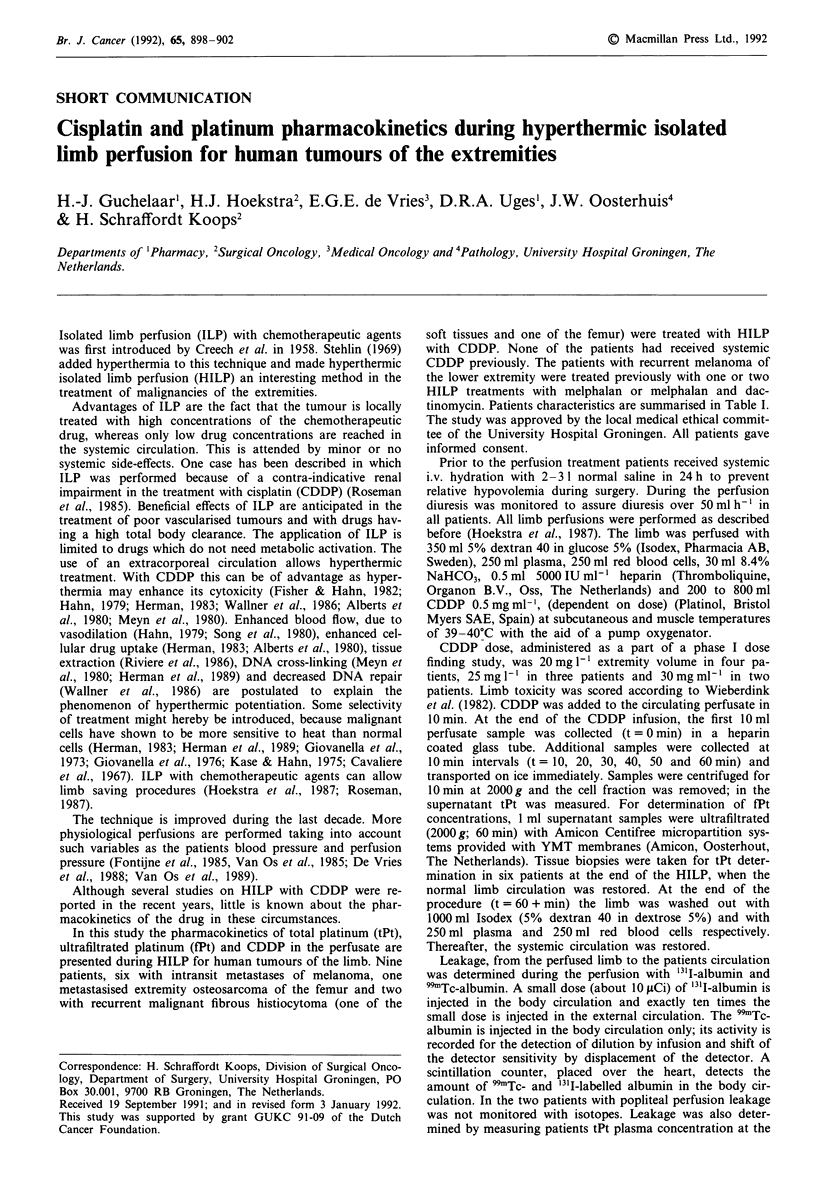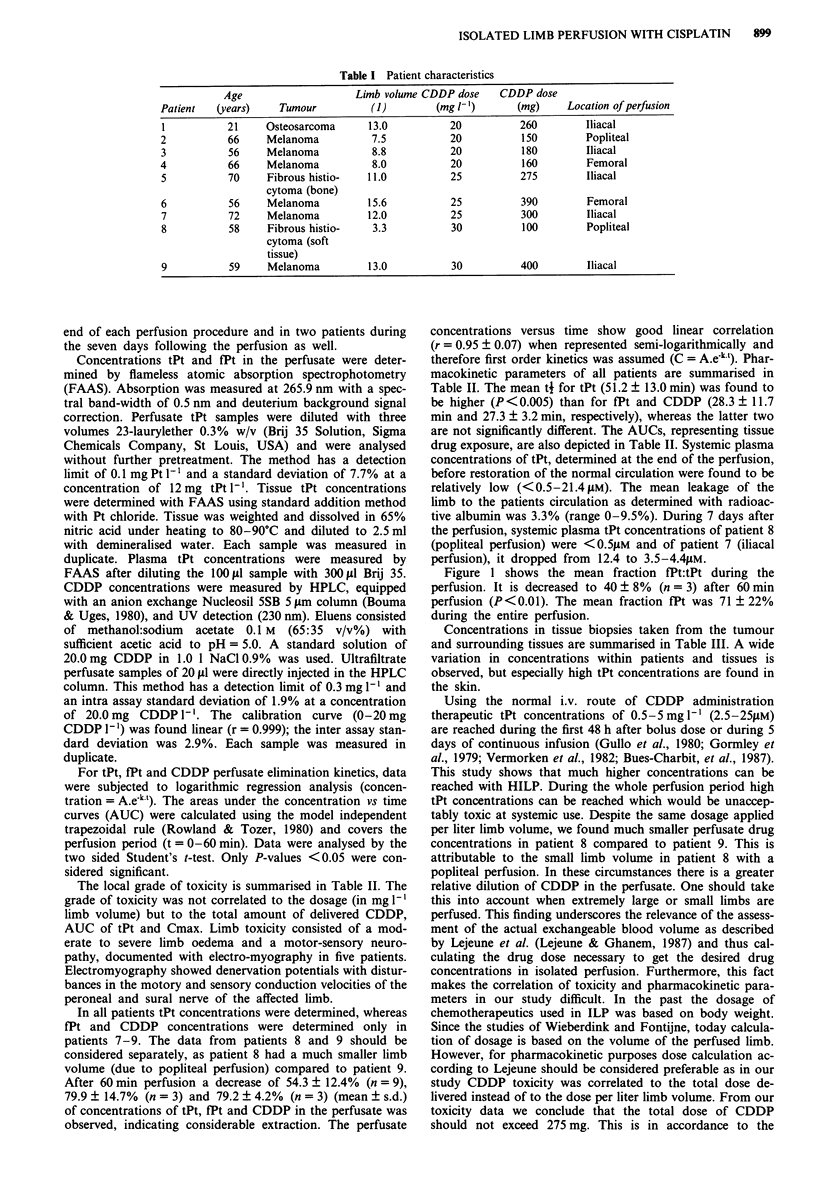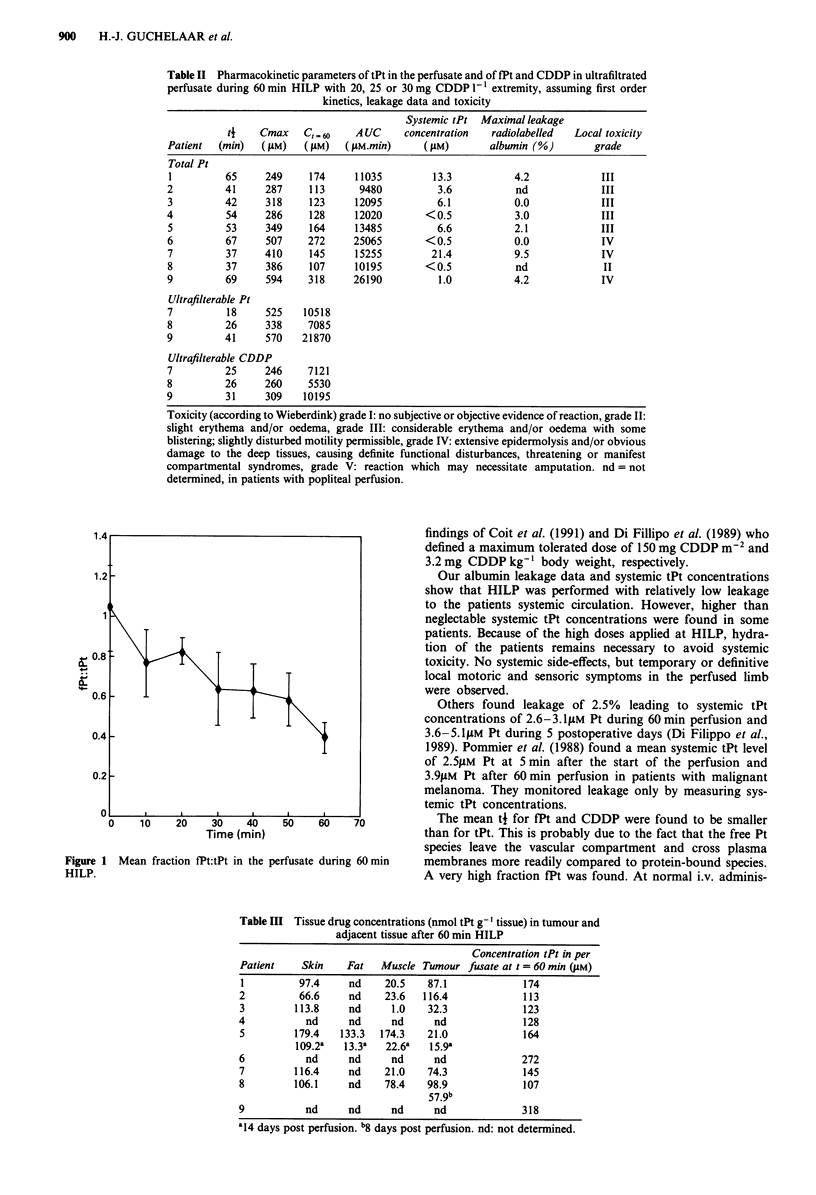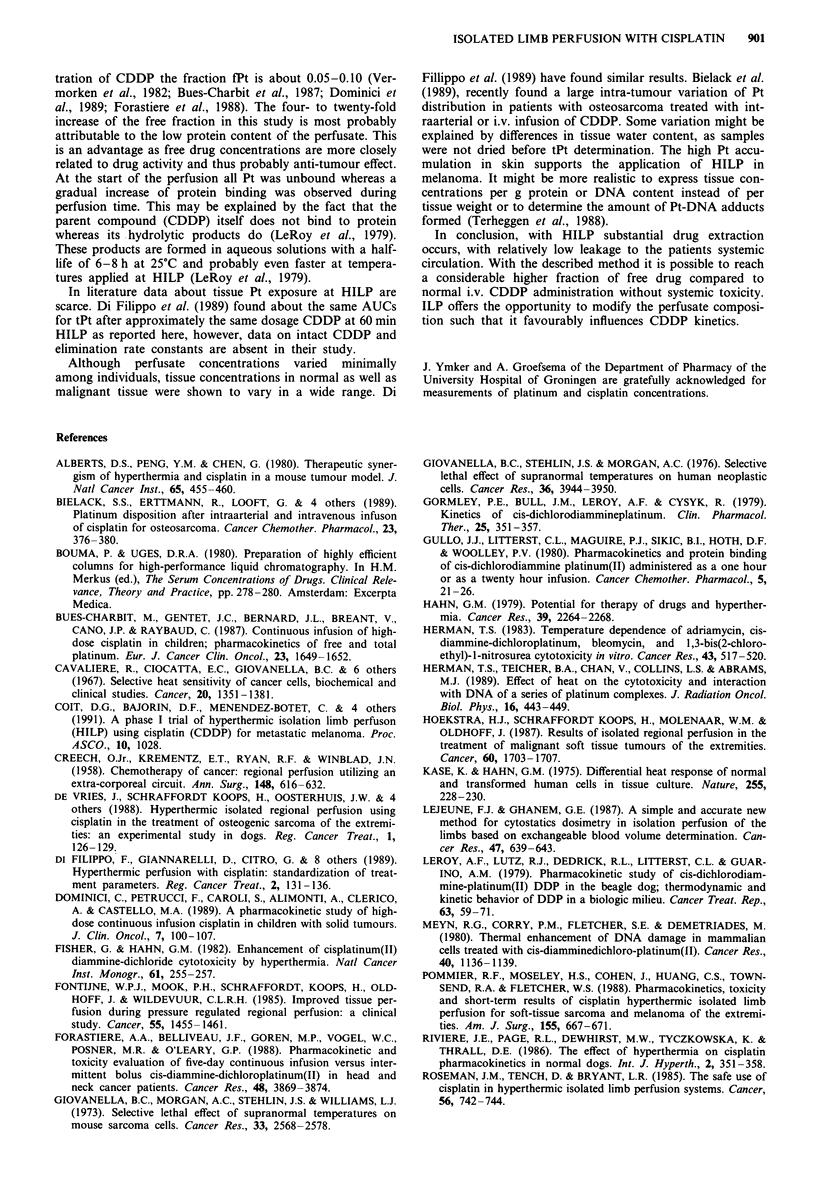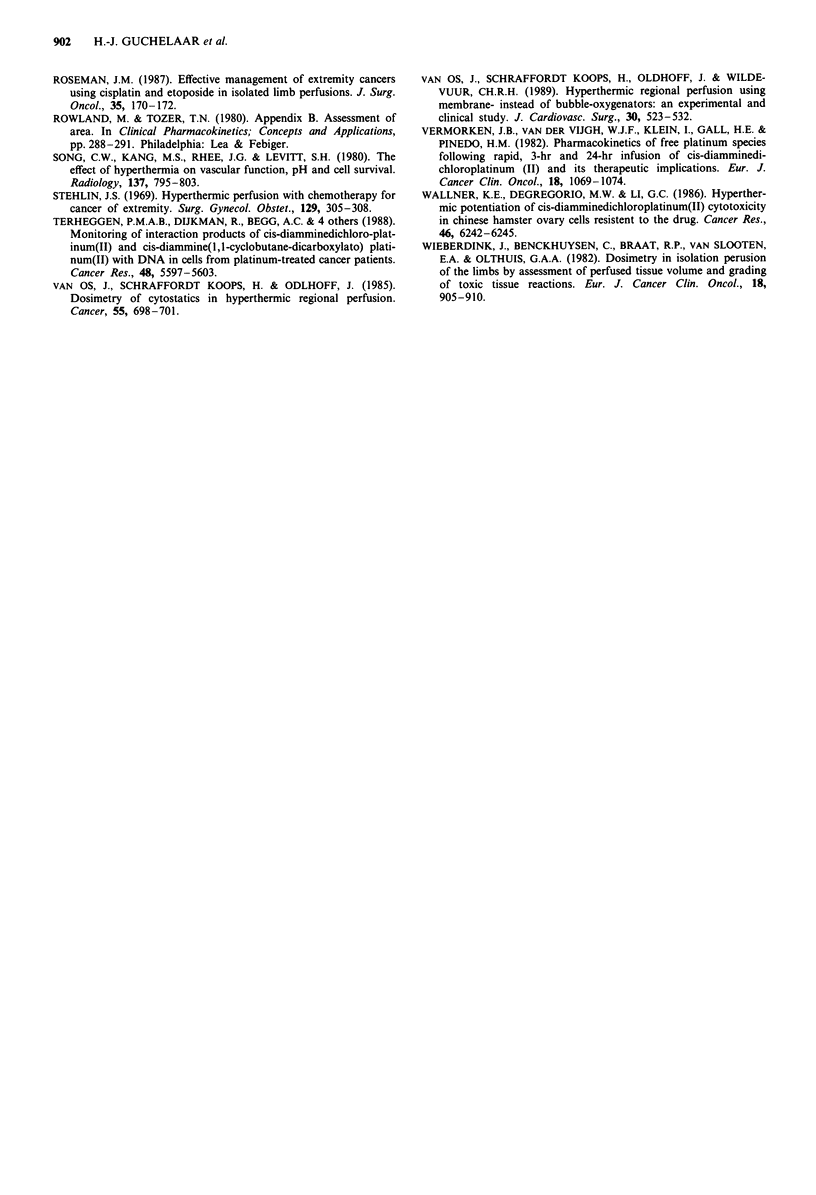# Cisplatin and platinum pharmacokinetics during hyperthermic isolated limb perfusion for human tumours of the extremities.

**DOI:** 10.1038/bjc.1992.188

**Published:** 1992-06

**Authors:** H. J. Guchelaar, H. J. Hoekstra, E. G. de Vries, D. R. Uges, J. W. Oosterhuis, H. Schraffordt Koops

**Affiliations:** Department of Pharmacy, University Hospital Groningen, The Netherlands.


Br. J. Cancer (1992), 65, 898-902 © Macmillan Press Ltd., 1992

SHORT COMMUNICATION

### Cisplatin and platinum pharmacokinetics during hyperthermic isolated limb perfusion for human tumours of the extremities

H.-J. Guchelaar[1], H.J. Hoekstra[2], E.G.E. de Vries[3], D.R.A. Uges[1], J.W. Oosterhuis[4] & H. Schraffordt Koops[2]

*Departments of [1]Pharmacy, [2]Surgical Oncology, [3]Medical Oncology and [4]Pathology, University Hospital Groningen, The Netherlands.*

Isolated limb perfusion (ILP) with chemotherapeutic agents was first introduced by Creech *et al.* in 1958. Stehlin (1969) added hyperthermia to this technique and made hyperthermic isolated limb perfusion (HILP) an interesting method in the treatment of malignancies of the extremities.

Advantages of ILP are the fact that the tumour is locally treated with high concentrations of the chemotherapeutic drug, whereas only low drug concentrations are reached in the systemic circulation. This is attended by minor or no systemic side-effects. One case has been described in which ILP was performed because of a contra-indicative renal impairment in the treatment with cisplatin (CDDP) (Roseman *et al.*, 1985). Beneficial effects of ILP are anticipated in the treatment of poor vascularised tumours and with drugs having a high total body clearance. The application of ILP is limited to drugs which do not need metabolic activation. The use of an extracorporeal circulation allows hyperthermic treatment. With CDDP this can be of advantage as hyperthermia may enhance its cytoxicity (Fisher & Hahn, 1982; Hahn, 1979; Herman, 1983; Wallner *et al.*, 1986; Alberts *et al.*, 1980; Meyn *et al.*, 1980). Enhanced blood flow, due to vasodilation (Hahn, 1979; Song *et al.*, 1980), enhanced cellular drug uptake (Herman, 1983; Alberts *et al.*, 1980), tissue extraction (Riviere *et al.*, 1986), DNA cross-linking (Meyn *et al.*, 1980; Herman *et al.*, 1989) and decreased DNA repair (Wallner *et al.*, 1986) are postulated to explain the phenomenon of hyperthermic potentiation. Some selectivity of treatment might hereby be introduced, because malignant cells have shown to be more sensitive to heat than normal cells (Herman, 1983; Herman *et al.*, 1989; Giovanella *et al.*, 1973; Giovanella *et al.*, 1976; Kase & Hahn, 1975; Cavaliere *et al.*, 1967). ILP with chemotherapeutic agents can allow limb saving procedures (Hoekstra *et al.*, 1987; Roseman, 1987).

The technique is improved during the last decade. More physiological perfusions are performed taking into account such variables as the patients blood pressure and perfusion pressure (Fontijne *et al.*, 1985, Van Os *et al.*, 1985; De Vries *et al.*, 1988; Van Os *et al.*, 1989).

Although several studies on HILP with CDDP were reported in the recent years, little is known about the pharmacokinetics of the drug in these circumstances.

In this study the pharmacokinetics of total platinum (tPt), ultrafiltrated platinum (fPt) and CDDP in the perfusate are presented during HILP for human tumours of the limb. Nine patients, six with intransit metastases of melanoma, one metastasised extremity osteosarcoma of the femur and two with recurrent malignant fibrous histiocytoma (one of the soft tissues and one of the femur) were treated with HILP with CDDP. None of the patients had received systemic CDDP previously. The patients with recurrent melanoma of the lower extremity were treated previously with one or two HILP treatments with melphalan or melphalan and dactinomycin. Patients characteristics are summarised in Table I. The study was approved by the local medical ethical committee of the University Hospital Groningen. All patients gave informed consent.

Prior to the perfusion treatment patients received systemic i.v. hydration with 2-3 l normal saline in 24 h to prevent relative hypovolemia during surgery. During the perfusion diuresis was monitored to assure diuresis over 50 ml $h^{-1}$ in all patients. All limb perfusions were performed as described before (Hoekstra *et al.*, 1987). The limb was perfused with 350 ml 5% dextran 40 in glucose 5% (Isodex, Pharmacia AB, Sweden), 250 ml plasma, 250 ml red blood cells, 30 ml 8.4% $NaHCO_3$, 0.5 ml 5000 IU $ml^{-1}$ heparin (Thromboliquine, Organon B.V., Oss, The Netherlands) and 200 to 800 ml CDDP 0.5 mg $ml^{-1}$, (dependent on dose) (Platinol, Bristol Myers SAE, Spain) at subcutaneous and muscle temperatures of 39-40°C with the aid of a pump oxygenator.

CDDP dose, administered as a part of a phase I dose finding study, was 20 mg $l^{-1}$ extremity volume in four patients, 25 mg $l^{-1}$ in three patients and 30 mg $ml^{-1}$ in two patients. Limb toxicity was scored according to Wieberdink *et al.* (1982). CDDP was added to the circulating perfusate in 10 min. At the end of the CDDP infusion, the first 10 ml perfusate sample was collected (t = 0 min) in a heparin coated glass tube. Additional samples were collected at 10 min intervals (t = 10, 20, 30, 40, 50 and 60 min) and transported on ice immediately. Samples were centrifuged for 10 min at 2000 *g* and the cell fraction was removed; in the supernatant tPt was measured. For determination of fPt concentrations, 1 ml supernatant samples were ultrafiltrated (2000 *g*; 60 min) with Amicon Centifree micropartition systems provided with YMT membranes (Amicon, Oosterhout, The Netherlands). Tissue biopsies were taken for tPt determination in six patients at the end of the HILP, when the normal limb circulation was restored. At the end of the procedure (t = 60 + min) the limb was washed out with 1000 ml Isodex (5% dextran 40 in dextrose 5%) and with 250 ml plasma and 250 ml red blood cells respectively. Thereafter, the systemic circulation was restored.

Leakage, from the perfused limb to the patients circulation was determined during the perfusion with $^{131}$I-albumin and $^{99m}$Tc-albumin. A small dose (about 10 μCi) of $^{131}$I-albumin is injected in the body circulation and exactly ten times the small dose is injected in the external circulation. The $^{99m}$Tc-albumin is injected in the body circulation only; its activity is recorded for the detection of dilution by infusion and shift of the detector sensitivity by displacement of the detector. A scintillation counter, placed over the heart, detects the amount of $^{99m}$Tc- and $^{131}$I-labelled albumin in the body circulation. In the two patients with popliteal perfusion leakage was not monitored with isotopes. Leakage was also determined by measuring patients tPt plasma concentration at the

Correspondence: H. Schraffordt Koops, Division of Surgical Oncology, Department of Surgery, University Hospital Groningen, PO Box 30.001, 9700 RB Groningen, The Netherlands.
Received 19 September 1991; and in revised form 3 January 1992.
This study was supported by grant GUKC 91-09 of the Dutch Cancer Foundation.

Table I Patient characteristics

| Patient | Age (years) | Tumour | Limb volume (l) | CDDP dose (mg l⁻¹) | CDDP dose (mg) | Location of perfusion |
|---|---|---|---|---|---|---|
| 1 | 21 | Osteosarcoma | 13.0 | 20 | 260 | Iliacal |
| 2 | 66 | Melanoma | 7.5 | 20 | 150 | Popliteal |
| 3 | 56 | Melanoma | 8.8 | 20 | 180 | Iliacal |
| 4 | 66 | Melanoma | 8.0 | 20 | 160 | Femoral |
| 5 | 70 | Fibrous histio-cytoma (bone) | 11.0 | 25 | 275 | Iliacal |
| 6 | 56 | Melanoma | 15.6 | 25 | 390 | Femoral |
| 7 | 72 | Melanoma | 12.0 | 25 | 300 | Iliacal |
| 8 | 58 | Fibrous histio-cytoma (soft tissue) | 3.3 | 30 | 100 | Popliteal |
| 9 | 59 | Melanoma | 13.0 | 30 | 400 | Iliacal |

end of each perfusion procedure and in two patients during the seven days following the perfusion as well.

Concentrations tPt and fPt in the perfusate were determined by flameless atomic absorption spectrophotometry (FAAS). Absorption was measured at 265.9 nm with a spectral band-width of 0.5 nm and deuterium background signal correction. Perfusate tPt samples were diluted with three volumes 23-laurylether 0.3% w/v (Brij 35 Solution, Sigma Chemicals Company, St Louis, USA) and were analysed without further pretreatment. The method has a detection limit of 0.1 mg Pt $l^{-1}$ and a standard deviation of 7.7% at a concentration of 12 mg tPt $l^{-1}$. Tissue tPt concentrations were determined with FAAS using standard addition method with Pt chloride. Tissue was weighted and dissolved in 65% nitric acid under heating to 80–90°C and diluted to 2.5 ml with demineralised water. Each sample was measured in duplicate. Plasma tPt concentrations were measured by FAAS after diluting the 100 µl sample with 300 µl Brij 35. CDDP concentrations were measured by HPLC, equipped with an anion exchange Nucleosil 5SB 5 µm column (Bouma & Uges, 1980), and UV detection (230 nm). Eluens consisted of methanol:sodium acetate 0.1 M (65:35 v/v%) with sufficient acetic acid to pH = 5.0. A standard solution of 20.0 mg CDDP in 1.0 l NaCl 0.9% was used. Ultrafiltrate perfusate samples of 20 µl were directly injected in the HPLC column. This method has a detection limit of 0.3 mg $l^{-1}$ and an intra assay standard deviation of 1.9% at a concentration of 20.0 mg CDDP $l^{-1}$. The calibration curve (0–20 mg CDDP $l^{-1}$) was found linear (r = 0.999); the inter assay standard deviation was 2.9%. Each sample was measured in duplicate.

For tPt, fPt and CDDP perfusate elimination kinetics, data were subjected to logarithmic regression analysis (concentration = $A.e^{-k.t}$). The areas under the concentration *vs* time curves (AUC) were calculated using the model independent trapezoidal rule (Rowland & Tozer, 1980) and covers the perfusion period (t = 0–60 min). Data were analysed by the two sided Student's *t*-test. Only *P*-values $<0.05$ were considered significant.

The local grade of toxicity is summarised in Table II. The grade of toxicity was not correlated to the dosage (in mg $l^{-1}$ limb volume) but to the total amount of delivered CDDP, AUC of tPt and Cmax. Limb toxicity consisted of a moderate to severe limb oedema and a motor-sensory neuropathy, documented with electro-myography in five patients. Electromyography showed denervation potentials with disturbances in the motory and sensory conduction velocities of the peroneal and sural nerve of the affected limb.

In all patients tPt concentrations were determined, whereas fPt and CDDP concentrations were determined only in patients 7–9. The data from patients 8 and 9 should be considered separately, as patient 8 had a much smaller limb volume (due to popliteal perfusion) compared to patient 9. After 60 min perfusion a decrease of 54.3 ± 12.4% ($n = 9$), 79.9 ± 14.7% ($n = 3$) and 79.2 ± 4.2% ($n = 3$) (mean ± s.d.) of concentrations of tPt, fPt and CDDP in the perfusate was observed, indicating considerable extraction. The perfusate concentrations versus time show good linear correlation ($r = 0.95 \pm 0.07$) when represented semi-logarithmically and therefore first order kinetics was assumed ($C = A.e^{-k.t}$). Pharmacokinetic parameters of all patients are summarised in Table II. The mean $t\frac{1}{2}$ for tPt (51.2 ± 13.0 min) was found to be higher ($P<0.005$) than for fPt and CDDP (28.3 ± 11.7 min and 27.3 ± 3.2 min, respectively), whereas the latter two are not significantly different. The AUCs, representing tissue drug exposure, are also depicted in Table II. Systemic plasma concentrations of tPt, determined at the end of the perfusion, before restoration of the normal circulation were found to be relatively low (<0.5–21.4 µM). The mean leakage of the limb to the patients circulation as determined with radioactive albumin was 3.3% (range 0–9.5%). During 7 days after the perfusion, systemic plasma tPt concentrations of patient 8 (popliteal perfusion) were <0.5µM and of patient 7 (iliacal perfusion), it dropped from 12.4 to 3.5–4.4µM.

Figure 1 shows the mean fraction fPt:tPt during the perfusion. It is decreased to 40 ± 8% ($n = 3$) after 60 min perfusion ($P<0.01$). The mean fraction fPt was 71 ± 22% during the entire perfusion.

Concentrations in tissue biopsies taken from the tumour and surrounding tissues are summarised in Table III. A wide variation in concentrations within patients and tissues is observed, but especially high tPt concentrations are found in the skin.

Using the normal i.v. route of CDDP administration therapeutic tPt concentrations of 0.5–5 mg $l^{-1}$ (2.5–25µM) are reached during the first 48 h after bolus dose or during 5 days of continuous infusion (Gullo *et al.*, 1980; Gormley *et al.*, 1979; Vermorken *et al.*, 1982; Bues-Charbit, *et al.*, 1987). This study shows that much higher concentrations can be reached with HILP. During the whole perfusion period high tPt concentrations can be reached which would be unacceptably toxic at systemic use. Despite the same dosage applied per liter limb volume, we found much smaller perfusate drug concentrations in patient 8 compared to patient 9. This is attributable to the small limb volume in patient 8 with a popliteal perfusion. In these circumstances there is a greater relative dilution of CDDP in the perfusate. One should take this into account when extremely large or small limbs are perfused. This finding underscores the relevance of the assessment of the actual exchangeable blood volume as described by Lejeune *et al.* (Lejeune & Ghanem, 1987) and thus calculating the drug dose necessary to get the desired drug concentrations in isolated perfusion. Furthermore, this fact makes the correlation of toxicity and pharmacokinetic parameters in our study difficult. In the past the dosage of chemotherapeutics used in ILP was based on body weight. Since the studies of Wieberdink and Fontijne, today calculation of dosage is based on the volume of the perfused limb. However, for pharmacokinetic purposes dose calculation according to Lejeune should be considered preferable as in our study CDDP toxicity was correlated to the total dose delivered instead of to the dose per liter limb volume. From our toxicity data we conclude that the total dose of CDDP should not exceed 275 mg. This is in accordance to the

**Table II** Pharmacokinetic parameters of tPt in the perfusate and of fPt and CDDP in ultrafiltrated perfusate during 60 min HILP with 20, 25 or 30 mg CDDP $l^{-1}$ extremity, assuming first order kinetics, leakage data and toxicity

| Patient | $t\frac{1}{2}$ (min) | Cmax (μM) | $C_{t=60}$ (μM) | AUC (μM.min) | Systemic tPt concentration (μM) | Maximal leakage radiolabelled albumin (%) | Local toxicity grade |
|---|---|---|---|---|---|---|---|
| *Total Pt* | | | | | | | |
| 1 | 65 | 249 | 174 | 11035 | 13.3 | 4.2 | III |
| 2 | 41 | 287 | 113 | 9480 | 3.6 | nd | III |
| 3 | 42 | 318 | 123 | 12095 | 6.1 | 0.0 | III |
| 4 | 54 | 286 | 128 | 12020 | <0.5 | 3.0 | III |
| 5 | 53 | 349 | 164 | 13485 | 6.6 | 2.1 | III |
| 6 | 67 | 507 | 272 | 25065 | <0.5 | 0.0 | IV |
| 7 | 37 | 410 | 145 | 15255 | 21.4 | 9.5 | IV |
| 8 | 37 | 386 | 107 | 10195 | <0.5 | nd | II |
| 9 | 69 | 594 | 318 | 26190 | 1.0 | 4.2 | IV |
| *Ultrafilterable Pt* | | | | | | | |
| 7 | 18 | 525 | 10518 | | | | |
| 8 | 26 | 338 | 7085 | | | | |
| 9 | 41 | 570 | 21870 | | | | |
| *Ultrafilterable CDDP* | | | | | | | |
| 7 | 25 | 246 | 7121 | | | | |
| 8 | 26 | 260 | 5530 | | | | |
| 9 | 31 | 309 | 10195 | | | | |

Toxicity (according to Wieberdink) grade I: no subjective or objective evidence of reaction, grade II: slight erythema and/or oedema, grade III: considerable erythema and/or oedema with some blistering; slightly disturbed motility permissible, grade IV: extensive epidermolysis and/or obvious damage to the deep tissues, causing definite functional disturbances, threatening or manifest compartmental syndromes, grade V: reaction which may necessitate amputation. nd = not determined, in patients with popliteal perfusion.

**Figure 1** Mean fraction fPt:tPt in the perfusate during 60 min HILP.

findings of Coit *et al.* (1991) and Di Fillipo *et al.* (1989) who defined a maximum tolerated dose of 150 mg CDDP $m^{-2}$ and 3.2 mg CDDP $kg^{-1}$ body weight, respectively.

Our albumin leakage data and systemic tPt concentrations show that HILP was performed with relatively low leakage to the patients systemic circulation. However, higher than neglectable systemic tPt concentrations were found in some patients. Because of the high doses applied at HILP, hydration of the patients remains necessary to avoid systemic toxicity. No systemic side-effects, but temporary or definitive local motoric and sensoric symptoms in the perfused limb were observed.

Others found leakage of 2.5% leading to systemic tPt concentrations of 2.6–3.1μM Pt during 60 min perfusion and 3.6–5.1μM Pt during 5 postoperative days (Di Filippo *et al.*, 1989). Pommier *et al.* (1988) found a mean systemic tPt level of 2.5μM Pt at 5 min after the start of the perfusion and 3.9μM Pt after 60 min perfusion in patients with malignant melanoma. They monitored leakage only by measuring systemic tPt concentrations.

The mean $t\frac{1}{2}$ for fPt and CDDP were found to be smaller than for tPt. This is probably due to the fact that the free Pt species leave the vascular compartment and cross plasma membranes more readily compared to protein-bound species. A very high fraction fPt was found. At normal i.v. adminis-

**Table III** Tissue drug concentrations (nmol tPt $g^{-1}$ tissue) in tumour and adjacent tissue after 60 min HILP

| Patient | Skin | Fat | Muscle | Tumour | Concentration tPt in perfusate at t = 60 min (μM) |
|---|---|---|---|---|---|
| 1 | 97.4 | nd | 20.5 | 87.1 | 174 |
| 2 | 66.6 | nd | 23.6 | 116.4 | 113 |
| 3 | 113.8 | nd | 1.0 | 32.3 | 123 |
| 4 | nd | nd | nd | nd | 128 |
| 5 | 179.4 | 133.3 | 174.3 | 21.0 | 164 |
| | 109.2[a] | 13.3[a] | 22.6[a] | 15.9[a] | |
| 6 | nd | nd | nd | nd | 272 |
| 7 | 116.4 | nd | 21.0 | 74.3 | 145 |
| 8 | 106.1 | nd | 78.4 | 98.9 | 107 |
| | | | | 57.9[b] | |
| 9 | nd | nd | nd | nd | 318 |

[a]14 days post perfusion. [b]8 days post perfusion. nd: not determined.

tration of CDDP the fraction fPt is about 0.05–0.10 (Vermorken *et al.*, 1982; Bues-Charbit *et al.*, 1987; Dominici *et al.*, 1989; Forastiere *et al.*, 1988). The four- to twenty-fold increase of the free fraction in this study is most probably attributable to the low protein content of the perfusate. This is an advantage as free drug concentrations are more closely related to drug activity and thus probably anti-tumour effect. At the start of the perfusion all Pt was unbound whereas a gradual increase of protein binding was observed during perfusion time. This may be explained by the fact that the parent compound (CDDP) itself does not bind to protein whereas its hydrolytic products do (LeRoy *et al.*, 1979). These products are formed in aqueous solutions with a half-life of 6–8 h at 25°C and probably even faster at temperatures applied at HILP (LeRoy *et al.*, 1979).

In literature data about tissue Pt exposure at HILP are scarce. Di Filippo *et al.* (1989) found about the same AUCs for tPt after approximately the same dosage CDDP at 60 min HILP as reported here, however, data on intact CDDP and elimination rate constants are absent in their study.

Although perfusate concentrations varied minimally among individuals, tissue concentrations in normal as well as malignant tissue were shown to vary in a wide range. Di Fillippo *et al.* (1989) have found similar results. Bielack *et al.* (1989), recently found a large intra-tumour variation of Pt distribution in patients with osteosarcoma treated with intraarterial or i.v. infusion of CDDP. Some variation might be explained by differences in tissue water content, as samples were not dried before tPt determination. The high Pt accumulation in skin supports the application of HILP in melanoma. It might be more realistic to express tissue concentrations per g protein or DNA content instead of per tissue weight or to determine the amount of Pt-DNA adducts formed (Terheggen *et al.*, 1988).

In conclusion, with HILP substantial drug extraction occurs, with relatively low leakage to the patients systemic circulation. With the described method it is possible to reach a considerable higher fraction of free drug compared to normal i.v. CDDP administration without systemic toxicity. ILP offers the opportunity to modify the perfusate composition such that it favourably influences CDDP kinetics.

J. Ymker and A. Groefsema of the Department of Pharmacy of the University Hospital of Groningen are gratefully acknowledged for measurements of platinum and cisplatin concentrations.

### References

ALBERTS, D.S., PENG, Y.M. & CHEN, G. (1980). Therapeutic synergism of hyperthermia and cisplatin in a mouse tumour model. *J. Natl Cancer Inst.*, **65**, 455–460.

BIELACK, S.S., ERTTMANN, R., LOOFT, G. & 4 others (1989). Platinum disposition after intraarterial and intravenous infuson of cisplatin for osteosarcoma. *Cancer Chemother. Pharmacol.*, **23**, 376–380.

BOUMA, P. & UGES, D.R.A. (1980). Preparation of highly efficient columns for high-performance liquid chromatography. In H.M. Merkus (ed.), *The Serum Concentrations of Drugs. Clinical Relevance, Theory and Practice*, pp. 278–280. Amsterdam: Excerpta Medica.

BUES-CHARBIT, M., GENTET, J.C., BERNARD, J.L., BREANT, V., CANO, J.P. & RAYBAUD, C. (1987). Continuous infusion of high-dose cisplatin in children; pharmacokinetics of free and total platinum. *Eur. J. Cancer Clin. Oncol.*, **23**, 1649–1652.

CAVALIERE, R., CIOCATTA, E.C., GIOVANELLA, B.C. & 6 others (1967). Selective heat sensitivity of cancer cells, biochemical and clinical studies. *Cancer*, **20**, 1351–1381.

COIT, D.G., BAJORIN, D.F., MENENDEZ-BOTET, C. & 4 others (1991). A phase I trial of hyperthermic isolation limb perfuson (HILP) using cisplatin (CDDP) for metastatic melanoma. *Proc. ASCO.*, **10**, 1028.

CREECH, O.Jr., KREMENTZ, E.T., RYAN, R.F. & WINBLAD, J.N. (1958). Chemotherapy of cancer: regional perfusion utilizing an extra-corporeal circuit. *Ann. Surg.*, **148**, 616–632.

DE VRIES, J., SCHRAFFORDT KOOPS, H., OOSTERHUIS, J.W. & 4 others (1988). Hyperthermic isolated regional perfusion using cisplatin in the treatment of osteogenic sarcoma of the extremities: an experimental study in dogs. *Reg. Cancer Treat.*, **1**, 126–129.

DI FILIPPO, F., GIANNARELLI, D., CITRO, G. & 8 others (1989). Hyperthermic perfusion with cisplatin: standardization of treatment parameters. *Reg. Cancer Treat.*, **2**, 131–136.

DOMINICI, C., PETRUCCI, F., CAROLI, S., ALIMONTI, A., CLERICO, A. & CASTELLO, M.A. (1989). A pharmacokinetic study of high-dose continuous infusion cisplatin in children with solid tumours. *J. Clin. Oncol.*, **7**, 100–107.

FISHER, G. & HAHN, G.M. (1982). Enhancement of cisplatinum(II) diammine-dichloride cytotoxicity by hyperthermia. *Natl Cancer Inst. Monogr.*, **61**, 255–257.

FONTIJNE, W.P.J., MOOK, P.H., SCHRAFFORDT, KOOPS, H., OLDHOFF, J. & WILDEVUUR, C.L.R.H. (1985). Improved tissue perfusion during pressure regulated regional perfusion: a clinical study. *Cancer*, **55**, 1455–1461.

FORASTIERE, A.A., BELLIVEAU, J.F., GOREN, M.P., VOGEL, W.C., POSNER, M.R. & O'LEARY, G.P. (1988). Pharmacokinetic and toxicity evaluation of five-day continuous infusion versus intermittent bolus cis-diammine-dichloroplatinum(II) in head and neck cancer patients. *Cancer Res.*, **48**, 3869–3874.

GIOVANELLA, B.C., MORGAN, A.C., STEHLIN, J.S. & WILLIAMS, L.J. (1973). Selective lethal effect of supranormal temperatures on mouse sarcoma cells. *Cancer Res.*, **33**, 2568–2578.

GIOVANELLA, B.C., STEHLIN, J.S. & MORGAN, A.C. (1976). Selective lethal effect of supranormal temperatures on human neoplastic cells. *Cancer Res.*, **36**, 3944–3950.

GORMLEY, P.E., BULL, J.M., LEROY, A.F. & CYSYK, R. (1979). Kinetics of cis-dichlorodiammineplatinum. *Clin. Pharmacol. Ther.*, **25**, 351–357.

GULLO, J.J., LITTERST, C.L., MAGUIRE, P.J., SIKIC, B.I., HOTH, D.F. & WOOLLEY, P.V. (1980). Pharmacokinetics and protein binding of cis-dichlorodiammine platinum(II) administered as a one hour or as a twenty hour infusion. *Cancer Chemother. Pharmacol.*, **5**, 21–26.

HAHN, G.M. (1979). Potential for therapy of drugs and hyperthermia. *Cancer Res.*, **39**, 2264–2268.

HERMAN, T.S. (1983). Temperature dependence of adriamycin, cis-diammine-dichloroplatinum, bleomycin, and 1,3-bis(2-chloroethyl)-1-nitrosurea cytotoxicity *in vitro*. *Cancer Res.*, **43**, 517–520.

HERMAN, T.S., TEICHER, B.A., CHAN, V., COLLINS, L.S. & ABRAMS, M.J. (1989). Effect of heat on the cytotoxicity and interaction with DNA of a series of platinum complexes. *J. Radiation Oncol. Biol. Phys.*, **16**, 443–449.

HOEKSTRA, H.J., SCHRAFFORDT KOOPS, H., MOLENAAR, W.M. & OLDHOFF, J. (1987). Results of isolated regional perfusion in the treatment of malignant soft tissue tumours of the extremities. *Cancer*, **60**, 1703–1707.

KASE, K. & HAHN, G.M. (1975). Differential heat response of normal and transformed human cells in tissue culture. *Nature*, **255**, 228–230.

LEJEUNE, F.J. & GHANEM, G.E. (1987). A simple and accurate new method for cytostatics dosimetry in isolation perfusion of the limbs based on exchangeable blood volume determination. *Cancer Res.*, **47**, 639–643.

LEROY, A.F., LUTZ, R.J., DEDRICK, R.L., LITTERST, C.L. & GUARINO, A.M. (1979). Pharmacokinetic study of cis-dichlorodiammine-platinum(II) DDP in the beagle dog; thermodynamic and kinetic behavior of DDP in a biologic milieu. *Cancer Treat. Rep.*, **63**, 59–71.

MEYN, R.G., CORRY, P.M., FLETCHER, S.E. & DEMETRIADES, M. (1980). Thermal enhancement of DNA damage in mammalian cells treated with cis-diamminedichloro-platinum(II). *Cancer Res.*, **40**, 1136–1139.

POMMIER, R.F., MOSELEY, H.S., COHEN, J., HUANG, C.S., TOWNSEND, R.A. & FLETCHER, W.S. (1988). Pharmacokinetics, toxicity and short-term results of cisplatin hyperthermic isolated limb perfusion for soft-tissue sarcoma and melanoma of the extremities. *Am. J. Surg.*, **155**, 667–671.

RIVIERE, J.E., PAGE, R.L., DEWHIRST, M.W., TYCZKOWSKA, K. & THRALL, D.E. (1986). The effect of hyperthermia on cisplatin pharmacokinetics in normal dogs. *Int. J. Hyperth.*, **2**, 351–358.

ROSEMAN, J.M., TENCH, D. & BRYANT, L.R. (1985). The safe use of cisplatin in hyperthermic isolated limb perfusion systems. *Cancer*, **56**, 742–744.

ROSEMAN, J.M. (1987). Effective management of extremity cancers using cisplatin and etoposide in isolated limb perfusions. *J. Surg. Oncol.*, **35**, 170–172.

ROWLAND, M. & TOZER, T.N. (1980). Appendix B. Assessment of area. In *Clinical Pharmacokinetics; Concepts and Applications*, pp. 288–291. Philadelphia: Lea & Febiger.

SONG, C.W., KANG, M.S., RHEE, J.G. & LEVITT, S.H. (1980). The effect of hyperthermia on vascular function, pH and cell survival. *Radiology*, **137**, 795–803.

STEHLIN, J.S. (1969). Hyperthermic perfusion with chemotherapy for cancer of extremity. *Surg. Gynecol. Obstet.*, **129**, 305–308.

TERHEGGEN, P.M.A.B., DIJKMAN, R., BEGG, A.C. & 4 others (1988). Monitoring of interaction products of cis-diamminedichloro-platinum(II) and cis-diammine(1,1-cyclobutane-dicarboxylato) platinum(II) with DNA in cells from platinum-treated cancer patients. *Cancer Res.*, **48**, 5597–5603.

VAN OS, J., SCHRAFFORDT KOOPS, H. & ODLHOFF, J. (1985). Dosimetry of cytostatics in hyperthermic regional perfusion. *Cancer*, **55**, 698–701.

VAN OS, J., SCHRAFFORDT KOOPS, H., OLDHOFF, J. & WILDEVUUR, CH.R.H. (1989). Hyperthermic regional perfusion using membrane- instead of bubble-oxygenators: an experimental and clinical study. *J. Cardiovasc. Surg.*, **30**, 523–532.

VERMORKEN, J.B., VAN DER VIJGH, W.J.F., KLEIN, I., GALL, H.E. & PINEDO, H.M. (1982). Pharmacokinetics of free platinum species following rapid, 3-hr and 24-hr infusion of cis-diamminedichloroplatinum (II) and its therapeutic implications. *Eur. J. Cancer Clin. Oncol.*, **18**, 1069–1074.

WALLNER, K.E., DEGREGORIO, M.W. & LI, G.C. (1986). Hyperthermic potentiation of cis-diamminedichloroplatinum(II) cytotoxicity in chinese hamster ovary cells resistent to the drug. *Cancer Res.*, **46**, 6242–6245.

WIEBERDINK, J., BENCKHUYSEN, C., BRAAT, R.P., VAN SLOOTEN, E.A. & OLTHUIS, G.A.A. (1982). Dosimetry in isolation perusion of the limbs by assessment of perfused tissue volume and grading of toxic tissue reactions. *Eur. J. Cancer Clin. Oncol.*, **18**, 905–910.